# DLCPD-25: A Large-Scale and Diverse Dataset for Crop Disease and Pest Recognition

**DOI:** 10.3390/s25227098

**Published:** 2025-11-20

**Authors:** Heng-Wei Zhang, Rui-Feng Wang, Zhengle Wang, Wen-Hao Su

**Affiliations:** 1 College of Engineering, China Agricultural University, 17 Qinghua East Road, Haidian, Beijing 100083, China; hwzhang@cau.edu.cn; 2 Department of Agricultural and Biological Engineering, University of Florida, Gainesville, FL 32611, USA; ruifeng.wang@ufl.edu; 3 Department of Crop and Soil Sciences, College of Agriculture and Environmental Sciences, University of Georgia, Tifton, GA 31793, USA; 4 College of Information and Electrical Engineering, China Agricultural University, 17 Qinghua East Road, Haidian, Beijing 100083, China; wangzhengle@cau.edu.cn

**Keywords:** self-supervised models, large-scale plant disease and pest dataset, disease and pest classification, self-supervised learning

## Abstract

The accurate identification of crop pests and diseases is critical for global food security, yet the development of robust deep learning models is hindered by the limitations of existing datasets. To address this gap, we introduce DLCPD-25, a new large-scale, diverse, and publicly available benchmark dataset. We constructed DLCPD-25 by integrating 221,943 images from both online sources and extensive field collections, covering 23 crop types and 203 distinct classes of pests, diseases, and healthy states. A key feature of this dataset is its realistic complexity, including images from uncontrolled field environments and a natural long-tail class distribution, which contrasts with many existing datasets collected under controlled conditions. To validate its utility, we pre-trained several state-of-the-art self-supervised learning models (MAE, SimCLR v2, MoCo v3) on DLCPD-25. The learned representations, evaluated via linear probing, demonstrated strong performance, with the SimCLR v2 framework achieving a top accuracy of 72.1% and an F1 score (Macro F1) of 71.3% on a downstream classification task. Our results confirm that DLCPD-25 provides a valuable and challenging resource that can effectively support the training of generalizable models, paving the way for the development of comprehensive, real-world agricultural diagnostic systems.

## 1. Introduction

Agricultural pests and diseases remain a major global challenge, threatening food security and economic stability [1,2]. According to the Food and Agriculture Organization of the United Nations (FAO) [3], such infestations cause up to 30% of annual crop yield losses and over 220 billion USD in direct economic damage. This situation underscores the urgent need for efficient monitoring and control systems. Early and accurate identification is essential for maintaining stable food production [4,5,6,7,8]. However, the vast diversity and morphological variability of pests and pathogens, which often differing markedly across developmental stages, make precise identification highly difficult [9]. Conventional detection methods rely on manual observation and expert visual inspection, which are time-consuming, labor-intensive, and inherently subjective. As a result, they fail to meet the efficiency requirements of modern large-scale agriculture, especially in extensive farmlands where early or localized outbreaks often go unnoticed [9,10].

To overcome these limitations, researchers began applying traditional machine learning (ML) techniques to pest and disease identification [11,12]. These approaches rely on manually designed features describing color, texture, and shape, which are then classified using models such as Support Vector Machines (SVM) and Random Forests (RF) [13]. For instance, spectral data collected by unmanned aerial vehicles (UAVs) have been used with SVM and RF classifiers to distinguish between healthy and aphid-infested wheat canopies, enabling threshold-based pest management [14,15]. Similar strategies employing multiclass SVMs have been applied for leaf segmentation and disease classification across multiple crops [14,16,17]. Other work combined computer vision techniques such as Histogram of Oriented Gradients (HOG) and K-means clustering with SVM classification to detect pests or leaf diseases with high accuracy [18,19]. Studies further highlight the utility of SVM, RF, and K-Nearest Neighbors (KNN) in assessing disease severity, such as potato late blight, from UAV imagery and spectral data [20,21,22]. Collectively, these studies demonstrated the feasibility of machine learning for automated pest and disease recognition and established the foundation for subsequent deep learning–based advancements.

Recent advances in deep learning and computer vision have opened new possibilities for precision agriculture, demonstrating exceptional potential in automated pest and disease identification [23,24]. Unlike traditional machine learning methods that rely on manually crafted features [6,25,26], Convolutional Neural Networks (CNNs) automatically learn hierarchical feature representations (from low-level textures to high-level semantic patterns) directly from raw images. This capacity substantially enhances model expressiveness, robustness, and generalization across varied agricultural conditions [27,28]. More recently, transformer-based architectures have emerged as a powerful alternative or complement to CNNs [29]. Through self-attention mechanisms, they capture long-range dependencies and global contextual relationships, enabling a more comprehensive understanding of spatial and structural information [30]. Such capabilities are particularly advantageous in complex agricultural environments characterized by cluttered backgrounds, occlusions, and high intra-class variability among pests and diseases [31,32]. Vision Transformers (ViTs) and hybrid transformer–CNN architectures have achieved state-of-the-art accuracy across multiple crop datasets while enabling real-time, end-to-end inference suitable for field deployment [33,34].

Numerous studies have demonstrated the powerful potential of deep learning in this field. Researchers have validated the application of CNNs in plant pathology tasks through various methods; for instance, Khan et al. [35] focused on optimizing a lightweight model (MobileNetV3-small) for edge computing devices, achieving 99.50% accuracy on the PlantVillage dataset. Concurrently, Babu et al. [36] achieved 96.99% accuracy in tomato disease detection by combining deep features from AlexNet, GoogleNet, and ResNet-50 and using an SVM for classification [35,36]. Subsequent research adopted deeper architectures such as VGG, ResNet, and Inception for pest and disease diagnosis [37]. For instance, Ferentinos [38] trained multiple CNN models on approximately 87,000 images and reached 99.53% accuracy across 58 categories, confirming the value of deep learning as a reliable early-warning tool [37,38]. These models not only achieve high accuracy across diverse disease types but also exhibit enhanced resilience to real-world variations in lighting, background complexity, and crop morphology.

Deep learning techniques have thus eliminated the dependence on manually designed features, enabling the automatic extraction of discriminative representations from large-scale image data through iterative optimization [39,40]. This capability has substantially enhanced robustness and recognition accuracy [41,42], establishing deep learning as the dominant paradigm for intelligent pest and disease identification.

The success of deep learning in this domain has been driven largely by the availability of several publicly released large-scale datasets that provide essential training and benchmarking resources. Among them, PlantVillage remains one of the most influential, containing over 50,000 leaf images captured under controlled conditions and covering 26 diseases across 14 crops [43]. For insect identification, the IP102 dataset offers a large-scale benchmark of 102 pest species, significantly advancing research on pest recognition [44]. Other datasets, including the New Plant Diseases Dataset [45] and CWD30 for crop–weed classification [46], further enrich the current data ecosystem for agricultural visual analysis. The emergence of these resources has markedly accelerated the application and development of deep learning techniques in precision agriculture.

Despite these advances, several key challenges persist, with dataset limitations remaining a central obstacle. This study therefore conducts an in-depth examination of existing agricultural pest and disease datasets and identifies four major issues.

1. Insufficient data scale and narrow category coverage. Early datasets, such as that of Prayma Bishshash [47], contain only 2137 images, far below the data requirements of modern deep learning models. Most existing datasets also focus on a limited set of common pests or diseases, whereas real agricultural environments involve hundreds of distinct species requiring recognition.
2. Simplified collection environments. More than 70% of available datasets are captured under controlled laboratory conditions, lacking realistic variations in illumination, occlusion, and soil backgrounds. Consequently, models trained on these datasets often achieve high laboratory accuracy but suffer substantial degradation when deployed in complex field settings [38].
3. Inadequate representation of intra-class variability and inter-class similarity. Pest and disease appearances can vary considerably across growth stages, plant conditions, and environmental contexts. Meanwhile, morphologically similar species, such as those within the IP102 dataset [44] or the grass-family species in CWD30 [46], remain difficult to distinguish. The absence of fine-grained annotations exacerbates this issue, reducing classification precision.
4. Class imbalance and annotation limitations. Pests and diseases in real fields follow long-tailed distributions, yet many datasets artificially resample data to balance categories. Although this simplifies training, it compromises the model’s ability to generalize to the true data distribution [48].

Despite the remarkable progress of deep learning in pest and disease identification, its performance still relies heavily on large volumes of high-quality, expert-annotated data [49,50]. In agriculture, obtaining such annotations is particularly challenging, as it demands specialized expertise in plant pathology and entomology, extensive labor, and significant financial resources [20]. The problem is further exacerbated by the long-tailed nature of agricultural datasets, where accurately labeling rare categories becomes especially demanding. These constraints hinder the construction of large-scale, representative, and scalable datasets essential for robust model training [2].

Self-supervised learning (SSL) offers a promising solution to this data bottleneck [51]. By leveraging vast amounts of unlabeled imagery, SSL enables models to learn transferable and semantically rich representations through pretext tasks such as predicting occluded regions or evaluating transformation consistency [52]. The representations learned in this manner can then be fine-tuned with limited labeled samples, often achieving or even surpassing the performance of fully supervised models [41,53].

Building on this paradigm, the central hypothesis of this study is that pretraining a deep model with SSL on a large and diverse dataset can produce a strong foundational visual model for agricultural applications. Such a model can substantially reduce dependence on manual annotation while enhancing adaptability and generalization in complex field environments.

To advance computer vision research in crop pest and disease recognition, this work introduces DLCPD-25 (Dataset of Large-scale Crop Pests and Diseases, 2025), comprising 221,943 images that encompass 203 pest, disease, and healthy categories across 23 crop species. The dataset exhibits a distinct long-tailed distribution and represents one of the largest and most diverse resources in the field. Its key advantages include:

1. Extensive coverage and large sample size. DLCPD-25 spans 23 major crops such as cotton, citrus, tomato, maize, soybean, grape, mango, wheat, sugar beet, apple, peach, rice, and alfalfa, containing 203 categories and over 221,000 images.
2. Inclusion of unlabeled field images for SSL validation. The dataset provides unlabeled samples for evaluating self-supervised frameworks.
3. Unified integration of pest, disease, and healthy samples. This design supports a transition from single-threat classification toward comprehensive diagnostic modeling for agricultural visual recognition.

Models pretrained on DLCPD-25 further validate its utility. The MAE model achieved 70.2% accuracy in cross-crop pest identification, while SimCLR v2 reached 72.1% accuracy and an F1-score of 71.3%. These results confirm that DLCPD-25 provides a solid foundation for developing annotation-efficient models capable of adapting to complex agricultural environments. Potential applications include UAV-based field monitoring and intelligent pest management in resource-limited regions.

The remainder of this paper is organized as follows: Section 2 describes the dataset construction process; Section 3 presents the results of unsupervised training and comparative analysis; Section 4 discusses distinctions between DLCPD-25 and mainstream agricultural datasets; Section 5 provides an extended discussion; and Section 6 concludes the study with a summary of its major contributions.

## 2. Construction of Proposed Dataset

Based on research experience, this study constructed and categorized the dataset through eight sequential stages: (1) online data collection; (2) field data acquisition; (3) data cleaning to remove low-quality images; (4) pest and disease identification by invited experts, assisted by volunteers in image recognition, classification, and screening; (5) establishment of a classification system; (6) preliminary categorization; (7) data augmentation; and (8) dataset partitioning. Among all collected data, 80.1% of the images were obtained from existing datasets, while 19.9% were collected through field sampling.

### 2.1. Online Collection and Curation

For the online data collection, this study conducted comprehensive searches across major data repositories and open-access platforms to acquire publicly available datasets relevant to plant disease and pest identification. The referenced datasets include the New Plant Diseases Dataset, IP102, and PlantVillage [43,44,45]. In selecting these sources, multiple factors were considered, including dataset provenance and scale, credibility, the number of plant disease or pest categories, and reported classification accuracy. In selecting these sources, our criteria were threefold, aligning with our primary goal of building a comprehensive pest and disease benchmark: (1) Thematic Relevance: The source must provide images of crop diseases or pests, not just healthy plants. (2) Novelty: The source should ideally contribute new disease or pest classes not already in our dataset. (3) Volume: The source could supplement the image count of existing classes to enhance diversity while maintaining the natural long-tail distribution. To ensure scientific rigor and reproducibility, only the training subsets of these open-source datasets were used, followed by systematic data cleaning. Low-quality and duplicate samples across different repositories were removed, and the curated data were subsequently organized into distinct groups for further processing.

Next, the collected datasets were reclassified and reorganized. The raw data obtained from online platforms exhibited substantial heterogeneity, manifested in three main aspects: (1) Inconsistent naming conventions: different data sources often used varying terminology for the same disease or pest, lacking a unified taxonomic standard; (2) Diverse classification hierarchies: discrepancies in labeling granularity across datasets increased the complexity of data integration; (3) Irrelevant content: some images did not focus on the target pests or diseases but instead depicted unrelated subjects. To address these issues, preliminary cleaning of the raw data was performed.

**Figure 1:**
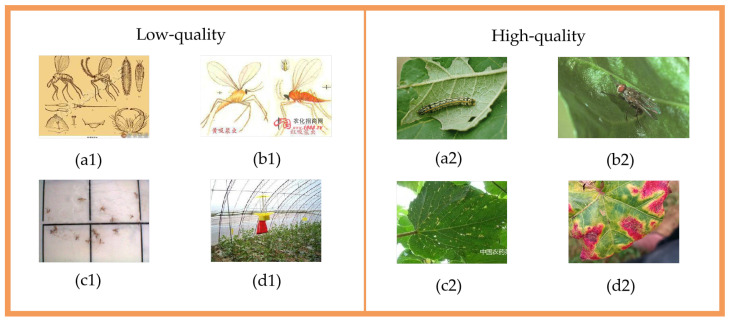
Examples of Low-quality Images (**a1**–**d1**) and High-quality Images (**a2**–**d2**) Online Open-sourced Datasets.

A rigorous multi-stage curation workflow was implemented to construct a standardized, high-quality dataset. Initially, naming inconsistencies were resolved using a unified ‘disease type–crop name’ schema. Domain experts in plant pathology and entomology then validated the taxonomy, merging semantically related categories to ensure scientific soundness. To eliminate redundancy, we employed the Perceptual Hashing (pHash) algorithm to detect and remove near-identical image fingerprints. Quality assessment followed a hybrid protocol: first, the Variance of the Laplacian algorithm (via OpenCV) was used to automatically screen out blurry images based on a variance threshold. Subsequently, trained volunteers inspected the data to exclude samples with poor exposure (over- or underexposure) and verified the visibility of target pests or diseases. This combination of automated and manual screening ensured that only clear, identifiable samples were retained. Representative examples of disqualified low-quality images contrasted with high-quality retained samples are shown in Figure 1, and through this systematic and expert-guided curation workflow, a refined and standardized online subset comprising 180,143 images consolidated into 192 distinct classes was ultimately established to support subsequent model training and evaluation.

### 2.2. In-Field Data Collection

To enhance the dataset’s practical applicability and generalization capacity, field-collected imagery was integrated to complement the open-source data. This large-scale, in-field acquisition aimed to address the limited environmental diversity and contextual realism of online datasets while expanding the overall scope and representativeness of the collection. To address the gaps identified in existing online datasets, we integrated field-collected imagery to complement the open-source data. Our preliminary analysis revealed two primary deficiencies in the online sources: inconsistent, and often low, image resolutions, and insufficient categorical coverage for key diseases in major crops.Therefore, our large-scale, in-field acquisition strategy was designed to address these specific gaps. Firstly, we aimed to expand the overall scope and representativeness by focusing on underrepresented categories. We specifically targeted 11 new disease and pest classes for critical crops like cotton, rice, and corn, which were absent from the curated online data. Secondly, to address quality and realism, we collected images under diverse field conditions (e.g., varying illumination, occlusion) while using a standardized capture device. This strategy not only supplements the dataset’s taxonomic coverage but also enhances its environmental diversity and practical applicability. Image acquisition was conducted at three experimental stations affiliated with China Agricultural University: the Zhuozhou Teaching and Experimental Farm (115.84° E, 39.47° N), the Quzhou Experimental Station (115.02° E, 36.86° N), and the Beijing Tongzhou Experimental Station (116.69° E, 39.70° N).

To ensure data consistency and reproducibility, all images were captured using a standardized handheld imaging device (model: “JIERUIWEITONG”), equipped with a 2.8 mm focal-length lens, a 90° field of view, and a resolution of 720p. The rationale for employing a consistent setup for our field sampling was to ensure this new data subset possessed high internal consistency and quality. The 720p resolution was chosen as a moderate balance: sufficient for potential future high-resolution studies yet not excessively divergent from the typical quality of the web-sourced data. The 2.8 mm focal length was selected for its wide field of view, which is practical for handheld field capture. During field collection, plant disease and pest samples were classified on-site, and preliminary identifications were immediately validated by agricultural experts to ensure accurate labeling and taxonomic reliability. This process initially yielded 59,800 candidate images representing a broad range of crops, disease symptoms, and pest conditions.

Subsequent quality assessment revealed that approximately 20% of these images exhibited deficiencies such as motion blur, defocused subjects, or the inclusion of irrelevant background elements (e.g., soil, sky, or shadows), primarily caused by handheld movement, variable lighting, or environmental disturbances. These low-quality samples were determined to be detrimental to model robustness and training efficiency and were therefore systematically excluded from the final dataset through a combination of automated screening and expert review.

### 2.3. Data Fusion, Filtering, and Annotation

**Figure 2:**
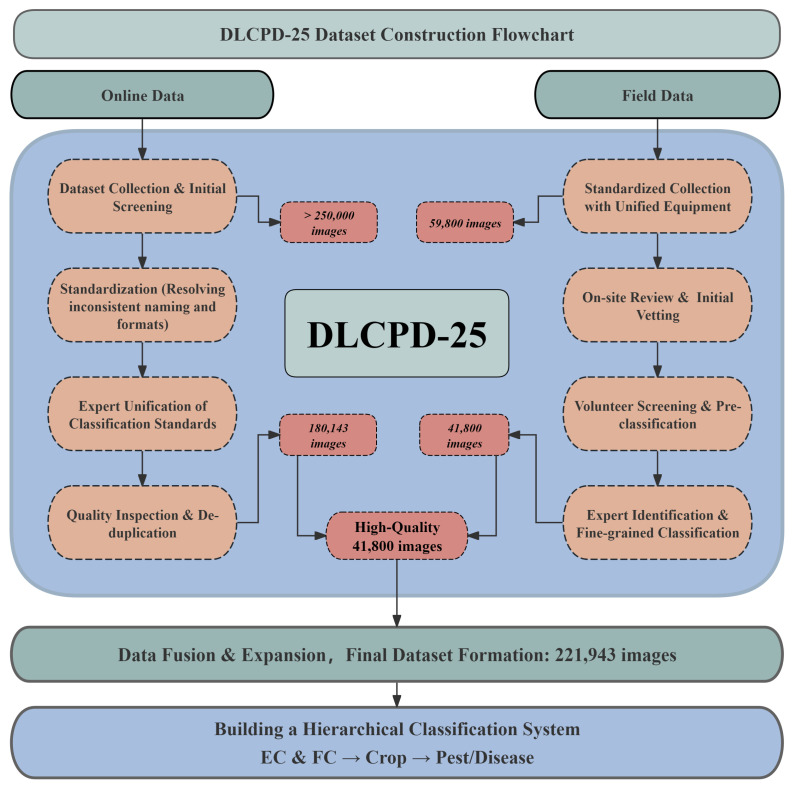
Workflow of dataset construction process.

To ensure the reliability and scientific integrity of the final dataset, a multi-stage data filtering, fusion, and annotation pipeline was implemented. The overall workflow for dataset construction is illustrated in Figure 2. Initially, a team of trained volunteers conducted a preliminary screening of the candidate images to eliminate samples severely affected by motion blur or containing irrelevant content. The filtered images were then subjected to coarse pre-classification based on their visual characteristics and collection locations. Finally, a comprehensive expert validation phase was carried out, during which specialists in plant pathology and entomology collaborated with the volunteers to perform definitive taxonomic verification, and final data consolidation, ensuring the accuracy and completeness of the dataset.

Through this rigorous multi-stage process, 41,800 high-quality in-field images were retained. These images were subsequently integrated with the curated online datasets to construct a unified and comprehensive collection. A portion of the field data was used to augment existing categories, thereby improving intra-class diversity and environmental robustness, while the remainder introduced new classes absent from the online sources, substantially expanding the dataset’s taxonomic coverage.

**Figure 3:**
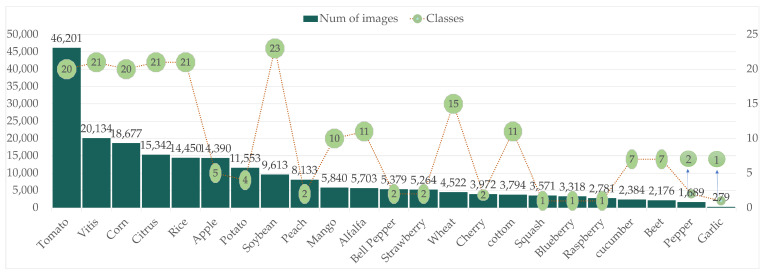
Sample distribution graph.

The resulting dataset, named DLCPD-25 (Dataset of Large-scale Crop Pests and Diseases, 2025), contains a total of 221,943 images encompassing 23 plant species and 210 distinct conditions, including pest infestations, disease symptoms, and healthy samples.The detailed information of the dataset can be found in Table A1. As depicted in Figure 3, the class distribution follows a typical long-tail pattern, reflecting the natural imbalance in agricultural ecosystems.

Following the data integration and refinement stages, a hierarchical classification system was established to organize the dataset systematically. The taxonomy is structured primarily by the host crop, with pests and diseases categorized as subclasses under their corresponding host species. At the highest level, all plant species are divided into two overarching groups: Economic Crops (EC) and Food Crops (FC). Within each group, every pest or disease instance is hierarchically nested under its host crop. For instance, Spodoptera litura, which primarily affects tomato plants, is categorized under “Tomato” within the broader EC class. This hierarchical design enhances interpretability and facilitates cross-crop comparative analyses. The overall dataset structure is summarized in Table 1.

**Figure 4:**
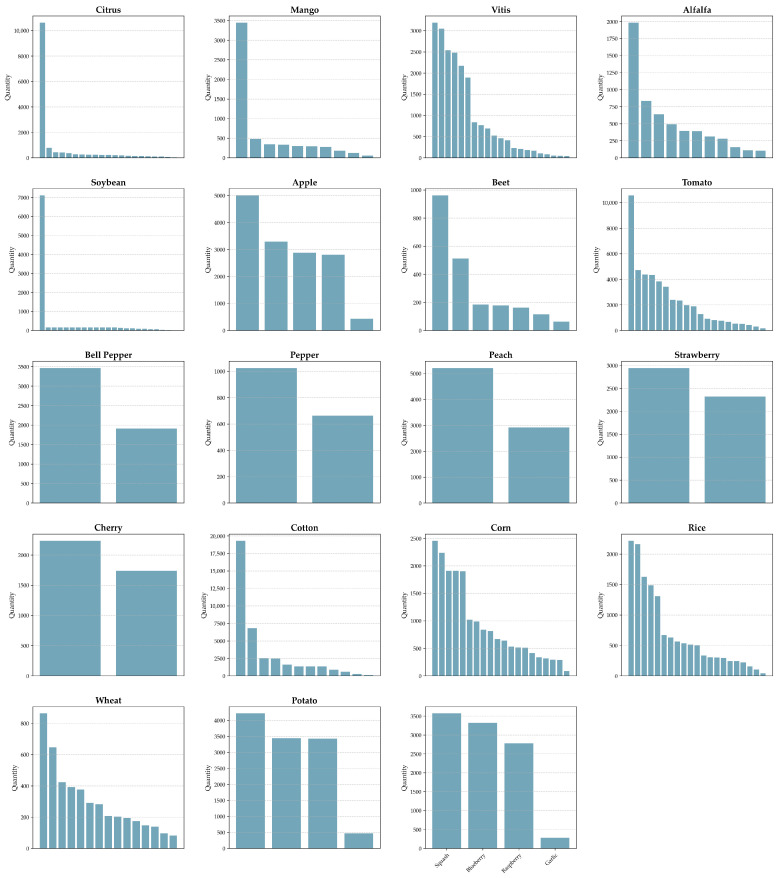
The distribution quantities of each category. In each crop-specific sub-plot, the x-axis represents distinct pest or disease classes sorted in descending order of sample size, and the y-axis represents the number of images.

Overall, the DLCPD-25 dataset provides a comprehensive and hierarchically structured resource for intelligent crop pest and disease detection. The EC category comprises 19 crop types (including tomato, cotton, cucumber, and apple) spanning 150 disease and health-related classes, whereas the FC category includes 4 staple crops (corn, rice, wheat, and potato) covering 60 distinct conditions. The distribution of samples across all categories is visualized in Figure 4, illustrating the dataset’s extensive coverage and diversity.

## 3. Comparative Analysis

### 3.1. Comparison with Other Datasets

To further emphasize the distinctive advantages of DLCPD-25 in terms of scale, diversity, and practical applicability, a comparative analysis was conducted against several representative agricultural datasets, including IP102 [44], CWD30 [46], and PDD271 [54]. All of these datasets are widely recognized within the field of agricultural computer vision and serve as important benchmarks for related research tasks. By comparing these representative datasets, the relative strengths and unique characteristics of DLCPD-25 can be demonstrated more clearly. The detailed comparison is presented in Table 2.

Based on the comparative results, DLCPD-25 shows distinct advantages across multiple dimensions, particularly in terms of dataset comprehensiveness and real-world representativeness. Its main strengths can be summarized as follows.

Leading Balance Between Scale and Category Diversity: DLCPD-25 achieves an optimal balance between dataset scale and categorical diversity. It contains 221,943 images, which is comparable to CWD30 (219,778) and the private PDD271 (220,592), and considerably larger than other well-known datasets such as PlantVillage (54,309) and IP102 (75,222). In terms of category count, DLCPD-25 includes 203 distinct classes, surpassing nearly all publicly available datasets and ranking just below PDD271, which remains inaccessible to the broader community. This extensive coverage provides a solid foundation for training deep learning models capable of recognizing a wide range of crop diseases and pests with improved generalization ability. Although IP102 includes pests from multiple crops, its classification scheme centers primarily on pest species rather than crop diversity [44], limiting its versatility for integrated diagnostic applications.

Comprehensive and Unique Coverage: DLCPD-25 distinguishes itself by offering the most comprehensive coverage among existing agricultural datasets. As illustrated in Table 2, most previous datasets focus on a single task dimension. For example, PlantVillage and PDDB target plant diseases, IP102 focuses exclusively on insect pests [44], and CWD30 is designed for crop–weed discrimination [46]. Models trained solely on such datasets often underperform in real-world agricultural scenarios, where diseases, pests, and weeds coexist. In contrast, DLCPD-25 is the first publicly available large-scale dataset to systematically integrate plant diseases, pest infestations, and healthy crop states within a unified framework. This comprehensive “integrated diagnostic” structure provides an essential basis for developing general-purpose agricultural recognition systems that align closely with actual field requirements. Such systems hold great potential for advancing precision agriculture, automated monitoring, and data-driven crop management.

High Scene Authenticity and Environmental Diversity: Another defining strength of DLCPD-25 lies in its authenticity and environmental diversity. Unlike datasets such as PlantVillage and PDDB, which were collected under controlled indoor conditions with uniform backgrounds, DLCPD-25 incorporates a wide variety of scenes captured both indoors and outdoors. The dataset includes images under complex illumination, occlusion, and background conditions, faithfully reflecting real-world variability. A substantial proportion of the data originates from field environments, containing realistic challenges such as uneven lighting, cluttered surroundings, and partial occlusions. Exposure to such diversity enables models trained on DLCPD-25 to develop inherent robustness and adaptability, which are critical for reliable performance in uncontrolled agricultural settings.

Open Accessibility and Research Value: Public availability is another key advantage of DLCPD-25. While datasets like PDD271 and IP102 show merit in certain metrics, their private nature restricts reproducibility and limits objective benchmarking across studies. DLCPD-25, in contrast, is fully open access, providing the global research community with a large-scale, high-quality benchmark for agricultural visual understanding. This openness fosters transparency, encourages fair comparison of emerging algorithms, and accelerates collaborative innovation within the field.

In summary, DLCPD-25 stands out among current agricultural datasets for its combination of large scale, extensive class diversity, comprehensive multi-domain coverage, and authentic environmental representation. Its open-access nature further enhances its scientific and practical value, positioning it as a cornerstone dataset for advancing intelligent, integrated, and field-oriented diagnostic systems in modern agriculture.

### 3.2. Other Potential Advantages

Closer Alignment with Real-World Data Distributions: The DLCPD-25 dataset exhibits a pronounced long-tailed distribution, which closely mirrors the naturally uneven occurrence frequencies of pests and diseases across real agricultural ecosystems. This characteristic presents inherent challenges for model optimization but simultaneously offers an opportunity for developing algorithms that are more resilient and adaptable to real-world variability [57]. By exposing models to data distributions that reflect practical agricultural conditions, DLCPD-25 encourages the creation of learning strategies capable of handling rare events and underrepresented classes, thus enhancing their robustness and ecological validity.

Integration of Self-Supervised Learning and Diagnostic Potential: A distinctive feature of DLCPD-25 lies in its inclusion of unlabeled field images and its demonstrated effectiveness when used within self-supervised learning frameworks. By validating the dataset through three representative self-supervised methods, this study highlights a promising pathway for utilizing large volumes of easily collected unlabeled data [58]. Such an approach can substantially reduce the reliance of agricultural vision systems on expensive manual annotations [59], paving the way toward scalable, cost-effective, and continuously evolving diagnostic models.

Unified Representation of Multiple Threat Types: DLCPD-25 integrates images of plant diseases, pest infestations, and healthy crops within a single dataset, facilitating a paradigm shift from isolated single-threat recognition toward comprehensive crop health diagnostics [60,61]. This integrated design differentiates DLCPD-25 from previous datasets that target narrowly defined tasks, such as IP102, which focuses solely on pest detection, and CWD30, which centers on weed recognition. Through this unified structure, DLCPD-25 provides a foundation for models that can jointly reason about multiple biological stressors, better reflecting the complexity of field conditions.

Foundation for Cross-Crop Pest Identification Research: During the dataset’s construction, particular emphasis was placed on capturing pest characteristics shared across different crop species. This characteristic positions DLCPD-25 as a valuable resource for advancing research into transferable and crop-agnostic diagnostic systems capable of adapting to novel species or regions.

In summary, DLCPD-25 demonstrates notable strengths in its scale, diversity, environmental authenticity, and integrated diagnostic orientation. By encompassing diverse crop species and biological conditions while incorporating both labeled and unlabeled data, it provides a comprehensive foundation for robust, intelligent, and annotation-efficient diagnostic systems. Its alignment with real-world data distributions and its demonstrated compatibility with self-supervised learning further enhance its practical relevance. Collectively, these attributes position DLCPD-25 as a transformative benchmark that bridges the gap between controlled laboratory research and real-world agricultural applications, guiding the evolution of computer vision in agriculture toward general, field-adaptive, and self-improving intelligent diagnostic systems.

## 4. Dataset Benchmarking

To comprehensively assess the feature extraction capabilities of various self-supervised learning (SSL) methods on the proposed DLCPD-25 dataset, a series of systematic benchmarking experiments were conducted. This section presents the experimental rationale, methodological framework, and performance evaluation criteria adopted in the analysis.

The choice of SSL as the core evaluation paradigm is motivated by a central challenge in agricultural computer vision—how to effectively exploit large-scale visual data that are often partially labeled or imperfectly annotated. Conventional supervised learning approaches rely heavily on extensive manual labeling and thus struggle to fully utilize datasets with long-tail class distributions, heterogeneous image qualities, and substantial proportions of unlabeled or weakly labeled samples [46]. These constraints limit their generalization and scalability when applied to real-world agricultural scenarios.

In contrast, SSL leverages intrinsic data regularities to learn meaningful visual representations without explicit reliance on class annotations. By modeling inherent patterns such as leaf venation structures, lesion texture gradients, or pest morphological cues, SSL methods can extract domain-relevant and semantically rich features directly from the image content. We hypothesize that pre-training on a large-scale, domain-specific dataset such as DLCPD-25 will yield feature representations that are more discriminative and contextually aligned with agricultural visual characteristics than those obtained from general-purpose datasets like ImageNet [58].

Accordingly, the principal objective of this benchmarking study is to identify the SSL strategy that most effectively captures transferable and robust features from DLCPD-25. The experimental results are intended to establish a standardized reference for future agricultural visual learning tasks and to demonstrate the dataset’s potential as a comprehensive pre-training resource for domain-adaptive model development.

### 4.1. Evaluation Method

This study adopts linear probing as the primary evaluation approach. Linear probing has become a standard and widely accepted method for assessing the quality of feature representations learned by self-supervised learning (SSL) models [62]. The central idea is to freeze the backbone network, which serves as the pretrained feature extractor [58], and then train only a lightweight linear classifier on top of it to perform downstream image classification. This protocol directly measures the linear separability of the learned features—that is, how well the representations can be distinguished using a simple linear mapping—thus providing a clear and interpretable indicator of feature quality [63].

To systematically evaluate the representational value of the proposed DLCPD-25 dataset, three milestone SSL frameworks were selected as benchmarks. These models are representative of the two dominant paradigms in self-supervised visual learning: contrastive learning and masked image modeling, both of which have achieved remarkable success in recent years due to their conceptual clarity and empirical performance.

#### 4.1.1. Masked Autoencoder

The Masked Autoencoder (MAE) [64] draws inspiration from the BERT architecture in natural language processing. The core idea is to partition an image into non-overlapping patches and randomly mask a large proportion of them, typically around 75%. MAE employs an asymmetric encoder–decoder architecture in which a ViT encoder processes only the visible patches to learn latent feature representations, while a lightweight decoder reconstructs the original image using the encoded features together with positional information from the masked patches [64].

This asymmetric design provides exceptional training efficiency. Because the encoder operates on only a subset of the input, computational and memory costs are substantially reduced compared with full-image processing. Such efficiency enables MAE to scale effectively to large model sizes and massive datasets [64,65,66]. Furthermore, by reconstructing missing content, the model learns high-level semantic representations that capture both object structure and global context rather than superficial textures. Owing to its simplicity, scalability, and strong performance across multiple visual tasks, MAE has rapidly become one of the most influential frameworks in modern visual self-supervised learning [65,67,68].

#### 4.1.2. SimCLR Series

SimCLR (Simple Framework for Contrastive Learning of Visual Representations) [58] represents a foundational step in contrastive visual learning. Its central principle is to apply two random data augmentations (such as random cropping, rotation, or color jittering) to the same image to form a positive pair [58,69]. Other samples within the same batch serve as negative pairs. The model is then trained to maximize the similarity between positive pairs while minimizing similarity with negatives in the feature space [58,70,71].

SimCLR v2 [72] builds upon this framework with several significant enhancements. It introduces deeper and wider backbone networks, expands the projection head to improve feature expressiveness, and explores a fine-tuning strategy for semi-supervised learning. In this strategy, only the first layer of the projection head is retained during fine-tuning, which leads to notable gains even when labeled data are limited.

One of SimCLR’s major strengths is its conceptual and architectural simplicity. It does not rely on complex mechanisms such as memory banks. Its effectiveness demonstrates that, when combined with strong data augmentation, nonlinear projection heads, and large-batch optimization, contrastive learning can yield robust and discriminative feature representations. Consequently, SimCLR and its improved variant have become canonical baselines for evaluating new SSL algorithms in both academic and industrial research [72].

#### 4.1.3. Momentum Contrast

The MoCo (Momentum Contrast) family [63] represents another major advancement in contrastive learning. To overcome SimCLR’s dependence on extremely large batch sizes for obtaining sufficient negative samples, early versions of MoCo introduced two key innovations: the momentum encoder and the dynamic dictionary queue. The queue stores a large and continuously updated set of negative samples far exceeding the batch size, while the momentum encoder—updated through an exponential moving average of the query encoder—ensures stable and consistent feature representations over time [63].

MoCo v3 [70] further refines this framework. It removes the dictionary queue in favor of large-batch training but retains the momentum encoder to stabilize ViT training, which is often unstable in self-supervised settings. An additional prediction head is also incorporated to further enhance convergence stability. By integrating the efficiency of SimCLR with the consistency mechanism of previous MoCo versions, MoCo v3 achieves strong robustness and scalability in ViT-based SSL pretraining [70]. It delivers state-of-the-art performance while maintaining high computational efficiency, making it one of the leading frameworks for large-scale visual representation learning.

**Figure 5:**
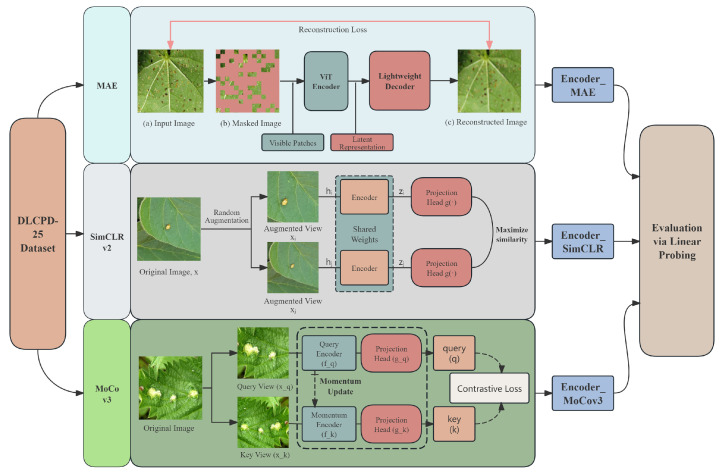
The main principles of the models and the evaluation process.

In summary, this study benchmarks three advanced SSL frameworks: MAE (a masked image modeling approach) [64], SimCLR v2 (an improved contrastive learning framework) [72], and MoCo v3 (a momentum-based contrastive learning framework) [70], on the DLCPD-25 dataset. Their core principles and the overall evaluation workflow adopted in this study are illustrated in Figure 5.

### 4.2. Evaluation Procedure

#### 4.2.1. Evaluation Protocol

To quantitatively evaluate the quality of feature representations learned by different self-supervised learning methods, this study adopts the academically recognized linear probing protocol as the primary evaluation strategy. Linear probing has been widely used to assess the linear separability and intrinsic quality of learned representations. To ensure full transparency and reproducibility, this section provides a detailed description of the experimental design, including dataset partitioning, implementation details, and the pretraining configurations of the SSL models.

#### 4.2.2. Dataset and Evaluation Setup

The DLCPD-25 dataset used in this study contains a total of 221,943 images. Using a fixed random seed, the dataset was divided into training and testing subsets in an approximate 80:20 ratio. Specifically, the training set includes 177,555 images, which were used exclusively for the self-supervised pretraining stage, while the testing set contains 44,388 images, reserved solely for evaluating the representations under the linear probing protocol. All images were uniformly resized to 256 × 256 pixels to ensure consistent input dimensions across all experiments.

In the evaluation stage, the backbone encoder of each pretrained model was frozen, and a single linear classifier was trained on top of the fixed representations using the training set. The classifier’s predictive performance was subsequently measured on the independent test set. This approach decouples representation quality from downstream fine-tuning complexity and directly reflects how well the extracted features can be separated through a simple linear mapping.

#### 4.2.3. Implementation Details and Pretraining Configuration

All experiments were conducted on a computing server equipped with two NVIDIA V100 GPUs (32 GB each). The software environment consisted of Ubuntu 21.04, Python 3.8, and the PyTorch 2.3.1 deep learning framework.

This study benchmarks three representative paradigms of self-supervised learning: Masked Image Modeling (MAE) and Contrastive Learning (SimCLR v2 and MoCo v3). The hyperparameters for each method were selected based on the best practices reported in their respective original publications, with appropriate adjustments to align with the DLCPD-25 dataset.

MAE: The Vision Transformer (ViT-Base) architecture was used as the backbone encoder. The model was pretrained on the DLCPD-25 training set for 1600 epochs with a batch size of 2048. The AdamW optimizer was employed, with a base learning rate (lr) of $1.5\times 10^{-4}$, scaled linearly according to Equation (1).

(1) $$\mathrm{lr}=\mathrm{base}\_\mathrm{lr}\times \frac{\mathrm{batch}\_\mathrm{size}}{256}$$

A cosine annealing learning rate schedule with 80 warm-up epochs was adopted. Following the original MAE design, 75% of the input patches were randomly masked, and minimal data augmentation was applied, consisting only of random resized cropping and horizontal flipping. This configuration preserves the semantic structure of the input while encouraging the model to learn holistic representations.

SimCLR v2: For contrastive learning, the ResNet-50 backbone was pretrained for 1600 epochs with a batch size of 4096. The LARS optimizer was used to accommodate large-batch training, and the learning rate was scaled according to Equation (2).

(2) $$\mathrm{lr}=0.3\times \frac{\mathrm{batch}\_\mathrm{size}}{256}$$

A cosine annealing schedule with warm-up was applied. To construct high-quality positive pairs, a strong data augmentation pipeline was used, including random resized cropping, horizontal flipping, color jittering (for brightness, contrast, saturation, and hue), random grayscale conversion, and Gaussian blurring. The temperature parameter in the InfoNCE loss was set to τ=0.1, and a three-layer MLP projection head was used to map feature embeddings into the contrastive space.

MoCo v3: In alignment with its native Vision Transformer design, MoCo v3 also adopted the ViT-Base architecture as its backbone network. The model was pretrained for 1000 epochs with a batch size of 2048. The AdamW optimizer was used with a base learning rate of $1.5\times 10^{-4}$, linearly scaled according to Equation (3).

(3) $$\mathrm{lr}=\mathrm{base}\_\mathrm{lr}\times \frac{\mathrm{batch}\_\mathrm{size}}{1024}$$

The learning rate followed a cosine decay schedule with warm-up. The same data augmentation settings as SimCLR v2 were applied to ensure comparable diversity among contrastive views. The momentum update coefficient for the momentum encoder was set to 0.99, while the temperature coefficient τ was fixed at 0.2, following the best practices for contrastive pretraining using Vision Transformers.

#### 4.2.4. Model Evaluation

After training the linear classifier, the model’s generalization performance was evaluated using previously unseen images from the DLCPD-25 test set $D_{\mathrm{test}}$. For each sample in the test set, the predicted class was recorded as $\hat{y}$. Two standard evaluation metrics were employed in this study:

Accuracy measures the proportion of correctly classified samples. It is defined as Equation (4)

(4) $$\mathrm{Accuracy}=\frac{1}{|D_{\mathrm{test}}|}\sum_{\left(x_{j},y_{j}\right)\in D_{\mathrm{test}}}1\left({\hat{y}}_{j}=y_{j}\right)$$

where $|D_{\mathrm{test}}|$ represents the total number of test samples, and 1(·) is the indicator function (equal to 1 if the condition holds, and 0 otherwise), and ${\hat{y}}_{j}$ denotes the predicted label for the test sample $x_{j}$.

Since accuracy alone can be misleading when dealing with imbalanced datasets, this study additionally adopts precision and recall, which provide a more detailed evaluation of model performance for each class. These metrics are based on the standard definitions of true positives (TP), false positives (FP), and false negatives (FN). For each class *c* in a multi-class setting, we define: ${\mathrm{TP}}_{c}$: the number of samples that belong to class *c* and are correctly predicted as *c*; ${\mathrm{FP}}_{c}$: the number of samples that do not belong to class *c* but are incorrectly predicted as *c*; ${\mathrm{FN}}_{c}$: the number of samples that belong to class *c* but are incorrectly predicted as another class. Based on these definitions, the precision and recall for each class are computed as Equations (5)–(8).

Precision ($P_{c}$): measures the proportion of correctly predicted samples among all samples predicted as class *c*, reflecting the reliability of predictions.

(5) $$P_{c}=\frac{{\mathrm{TP}}_{c}}{{\mathrm{TP}}_{c}+{\mathrm{FP}}_{c}}$$

Recall ($R_{c}$): measures the proportion of correctly predicted samples among all true samples of class *c*, reflecting the model’s ability to detect that class.

(6) $$R_{c}=\frac{{\mathrm{TP}}_{c}}{{\mathrm{TP}}_{c}+{\mathrm{FN}}_{c}}$$

Macro F1 Score (Macro F1): To assess the overall performance of the model under class imbalance, the macro-averaged F1 score (Macro F1) was computed.

For each class *c*, the F1 score ($F1_{c}$) is first defined as Equation (7).

(7) $$F1_{c}=2\times \frac{P_{c}\times R_{c}}{P_{c}+R_{c}}$$

Finally, to evaluate overall performance across all classes under imbalance conditions, the macro-averaged F1 score (Macro F1) is calculated as Equation (8).

(8) $$\mathrm{Macro}\;F1=\frac{1}{C}\sum_{c=1}^{C}F1_{c}$$

### 4.3. Results

Model performance was evaluated using two principal metrics: accuracy and the F1 score (Macro F1) [73]. Accuracy measures the proportion of correctly classified samples among all test samples, providing a direct and intuitive indicator of the overall predictive capability. In contrast, the F1 score ($F1_{c}$), which is defined as the harmonic mean of precision and recall, offers a more balanced evaluation, particularly under conditions of class imbalance or when both false positive and false negative rates are critical to performance assessment [74].

The quantitative results of the linear probing experiments on the DLCPD-25 dataset are summarized in Table 3.

Among the three self-supervised learning frameworks, SimCLR v2 achieved the highest accuracy and F1 score (Macro F1), reaching 72.1% and 71.3%, respectively. This indicates that the contrastive learning approach employed by SimCLR v2 effectively captures discriminative and transferable features from the agricultural imagery in DLCPD-25. The MoCo v3 model achieved comparable performance, demonstrating that the momentum-based contrastive learning mechanism can also extract robust representations under large-scale agricultural data conditions. The MAE model, based on masked image modeling, exhibited slightly lower accuracy and F1 score (Macro F1) (70.2% and 69.9%), suggesting that, although it learns rich contextual representations, its performance in linear separability may be somewhat constrained without task-specific fine-tuning.

Overall, the results validate that contrastive learning methods demonstrate superior linear separability and representation transferability compared with masked image modeling approaches when trained on DLCPD-25. This outcome highlights the dataset’s potential as a benchmark resource for developing and evaluating advanced self-supervised visual representation models tailored to agricultural scenarios.

## 5. Discussion

This study constructed and released DLCPD-25, a large-scale, highly diverse, and field-realistic dataset for crop pest and disease identification. The dataset contains 221,943 images covering 203 conditions across 23 crop species and integrates both online and field-collected data while preserving the long-tailed distribution inherent to real agricultural ecosystems. Experimental results demonstrate that self-supervised models pretrained on DLCPD-25, such as SimCLR v2 and MAE, can effectively learn discriminative visual representations and achieve strong performance in cross-crop pest recognition tasks. These findings validate the dataset’s potential to support the development of more robust and efficient intelligent recognition systems for agricultural applications.

### 5.1. Advantages

The primary strengths of this study lie in its comprehensiveness and authenticity. Unlike most existing datasets collected under controlled laboratory conditions or focused narrowly on a single type of biological stressor, DLCPD-25 achieves a large-scale and systematic integration of crop disease, pest, and healthy samples. This integrated diagnostic perspective better aligns with the complexity of real agricultural production and promotes a transition from isolated “single-threat classification” to a holistic framework for crop health assessment.

Another notable advantage of DLCPD-25 is its retention of the long-tailed data distribution that characterizes real-world agricultural environments. Although this inherent imbalance introduces challenges during model optimization, it enables trained models to develop stronger generalization capabilities and improved adaptability to naturally uneven pest and disease occurrences. Consequently, models trained on DLCPD-25 are more likely to maintain stability and accuracy when applied to practical agricultural monitoring scenarios.

### 5.2. Challenges

Despite its contributions, several limitations should be acknowledged. First, although the dataset includes multiple crops and regions, the geographical scope and environmental variability of data collection remain limited, which may introduce regional biases. Pest and disease manifestations can vary significantly across different climatic and soil conditions, and these variations are not yet fully represented.

Second, DLCPD-25 consists primarily of static imagery, which does not capture temporal dynamics that could describe the progression and interaction of pest and disease development over time. Temporal continuity is an essential factor for studying outbreak prediction and life-cycle modeling.

Finally, although expert review was incorporated during annotation, the labeling granularity could be further refined. Future versions of DLCPD-25 may include additional metadata such as disease severity levels, pest developmental stages, and symptom progression patterns. These enhancements would allow for deeper model interpretability and more nuanced agricultural decision making.

### 5.3. Future Perspectives

Looking forward, future research based on DLCPD-25 can evolve along several promising directions:

1. Field deployment and validation: A key objective for future work is the deployment of DLCPD-25-trained models on edge-computing platforms, including drones, field robots, and mobile devices, to realize automated and real-time field monitoring systems [75]. Achieving this will require model compression, quantization, and architecture optimization to meet hardware constraints, as well as solutions for handling field-specific challenges such as motion blur, illumination changes, and target occlusion in dynamic environments.
2. Data augmentation via generative AI (GenAI): To mitigate data scarcity for rare pest and disease classes and to expand coverage under extreme environmental conditions (such as drought, flooding, or frost), future studies may employ advanced generative artificial intelligence techniques, including diffusion models [76,77] and generative adversarial networks (GANs) [78,79]. These methods can synthesize high-quality and diverse imagery to supplement underrepresented categories and rare scenarios, thereby improving both model robustness and dataset completeness. Furthermore, the integration of digital twin technologies could enable the generation of physically consistent virtual crop environments [80,81], facilitating dynamic and controllable simulation of pest and disease progression under varying climatic and management conditions.
3. Multimodal data fusion: In addition to visual imagery, integrating DLCPD-25 with multimodal data—such as meteorological variables, soil sensor measurements, and hyperspectral or multispectral imaging—could further enhance diagnostic precision and predictive capability [82]. Such integration would allow for a deeper understanding of crop–environment interactions and support the development of intelligent decision-support systems for precision agriculture.
4. Comprehensive comparative benchmarking: While this study validated the effectiveness of DLCPD-25 as a pre-training resource, a valuable future study would involve a direct, large-scale comparative experiment against other public datasets, such as PlantVillage and CWD30. Training identical SSL models on these different datasets and evaluating them on a standardized, unseen test set would provide definitive quantitative insights into the practical advantages of DLCPD-25’s scale, diversity, and field-realism, which we have identified as a priority for our ongoing research.

In conclusion, the construction of DLCPD-25 represents a significant step forward in agricultural artificial intelligence. The dataset provides a foundational resource for developing intelligent, robust, and practical pest and disease diagnostic systems that are more closely aligned with real-world conditions. Through its scale, diversity, and adaptability to self-supervised learning paradigms, DLCPD-25 offers a platform for future innovations that bridge the gap between controlled laboratory research and the complex realities of field applications. It is expected to play a pivotal role in advancing agricultural computer vision from task-specific recognition toward more general, adaptive, and sustainable intelligent diagnostic frameworks.

## 6. Conclusions

To overcome the limitations of existing agricultural pest and disease datasets in terms of scale, diversity, and real-world applicability, this study constructed a large-scale, high-quality benchmark dataset named DLCPD-25. Following systematic data cleaning and expert validation, DLCPD-25 contains 221,943 images encompassing 203 categories across 23 major crops, including both healthy and diseased states. The dataset integrates web-sourced and field-collected imagery while maintaining the inherent long-tailed distribution characteristic of natural agricultural environments. This design represents a conceptual shift from conventional “single-threat classification” toward the creation of a comprehensive crop health diagnostic framework that better reflects field realities.

By employing several state-of-the-art self-supervised learning frameworks, including Masked Autoencoders (MAEs), SimCLR v2, and MoCo v3, the effectiveness of DLCPD-25 was systematically validated. The experimental results showed that models pretrained on DLCPD-25 achieved up to 72.1% accuracy and a 71.3% F1 score (Macro F1) in downstream cross-crop pest recognition tasks. These findings demonstrate that DLCPD-25 enables the learning of rich, discriminative, and transferable visual representations while also revealing its potential to support efficient model training with large volumes of unlabeled agricultural data.

In conclusion, DLCPD-25 provides not only a valuable dataset for academic research but also a foundational platform for the development of intelligent and cost-efficient diagnostic systems, including drone-based field monitoring and automated detection applications. Through its scale, diversity, and authenticity, DLCPD-25 lays the groundwork for advancing agricultural computer vision toward more generalizable, adaptive, and intelligent crop health management solutions that are capable of meeting the practical demands of modern precision agriculture.

## Figures and Tables

**Table 1:** Hierarchical structure of the DLCPD-25 dataset.

Type	Crop Name	Classes	Num of Images
EC	Citrus	21	15,342
	Tomato	20	46,201
	Vitis	21	20,134
	Apple	5	14,390
	Soybean	23	9613
	Peach	2	8133
	Mango	10	5840
	Alfalfa	11	5703
	Bell Pepper	2	5379
	Strawberry	2	5264
	Cherry	2	3972
	Cotton	11	3794
	Squash	1	3571
	Blueberry	1	3318
	Raspberry	1	2781
	Cucumber	7	2384
	Beet	7	2176
	Pepper	2	1689
	Garlic	1	279
FC	Corn	20	18,677
	Rice	21	14,450
	Potato	4	11,553
	Wheat	15	4522

**Table 2:** Comparison of DLCPD-25 with other representative agricultural datasets.

Dataset	Image Count	Category Count	Coverage	Availability	Reference	Main Task
PDDB	46,409	56	Crop and Fruit Diseases	Public	[55]	Image classification
CWD30	219,778	30	Weeds	Public	[46]	Image classification
Plant Village	54,309	38	Crop and Fruit Diseases	Public	[43]	Image classification
Plant Doc	2598	17	Crop and Fruit Diseases	Public	[56]	Image classification and object detection
PDD271	220,592	271	Crop and Fruit Diseases	Private	[54]	Image classification
IP102	75,222	102	Pests	Private	[44]	Image classification and object detection
DLCPD-25	221,943	203	Diseases and Pests	Public	Ours	Image classification

DLCPD-25 exhibits the largest image scale (Image Count) and shows high diversity in coverage and category count.

**Table 3:** Linear probing results of different self-supervised models on the DLCPD-25 dataset.

Method	Accuracy (%)	F1 Score (%)	Precision (%)	Recall (%)
MAE	70.2	69.9	72.0	68.0
SimCLR v2	72.1	71.3	74.0	69.0
MoCo v3	71.2	70.4	73.0	68.0

All models were evaluated using the linear probing protocol on the DLCPD-25 dataset.

## Data Availability

The DLCPD-25 dataset introduced and analyzed in this study is publicly available at: https://github.com/hwzhanng/DLCPD-25-Dataset (accessed on 20 October 2025). The repository provides access to all image data, and relevant documentation used in this research.

## Appendix A. Complete List of Categories

The following table provides a detailed list of all categories included in the dataset, including species classification, scientific names, and sample quantities.

**Table A1:** List of detected species in the dataset.

Species	Scientific Name	Quantity	Num
Citrus	*Adristyrannus(Citrus)*	186	1
	*Aleurocanthus Spiniferus(Citru)*	414	2
	*Aphis Citricola Vander Goot(Citru)*	210	3
	*Bactrocera Tsuneonis(Citru)*	100	4
	*Ceroplastes Rubens(Citru)*	154	5
	*Chinese Citrus Fly(Citru)*	232	6
	*Chrysomphalus Aonidum(Citru)*	135	7
	*Dacus Dorsalis(Hendel)(Citru)*	263	8
	*Icerya Purchasi Maskell(Citru)*	433	9
	*Nipaecoccus Vastalor(Citru)*	59	10
	*Orange Huanglongbing(Citrus Greening)*	10,619	11
	*Panonchus Citri McGregor(Citru)*	231	12
	*Papilio Xuthus(Citru)*	269	13
	*Parlatoria Zizyphus Lucus(Citru)*	44	14
	*Phyllocnistis Citrella Stainton(Citru)*	242	15
	*Phyllocoptes Oleiverus Ashmead(Citru)*	103	16
	*Prodenia Litura(Citru)*	782	17
	*Toxoptera Aurantii(Citru)*	135	18
	*Toxoptera Citricidus(Citru)*	113	19
	*Unaspis Yanonensis(Citru)*	251	20
	*Citrus Healthy*	367	21
Mango	*Chlumetia Transversa(Mango)*	183	1
	*Cicadellidae(Mango)*	3444	2
	*Deporaus Marginatus Pascoe(Mango)*	199	3
	*Drosophila Melanogaster(Mango)*	740	4
	*Erosomyia Mangicola Shi(Mango)*	224	5
	*Idioscopus Clypealis(Lethierry)(Mango)*	424	6
	*Parasa Lepida(Mango)*	213	7
	*Scirtothrips Dorsalis Hood(Mango)*	399	8
	*Sternochetus Frigidus(Mango)*	187	9
	*Mango Anthracnose*	513	10
	*Mango Flat Beak Leafhopper(Mango)*	260	11
	*Mango Healthy*	229	12
Vitis	*Ampelophaga Rubiginosa(Vitis)*	458	1
	*Colomerus Vitis(Vitis)*	317	2
	*Erythroneura Apicalis(Vitis)*	323	3
	*Lycorma Delicatula(Vitis)*	218	4
	*Nipponaphis(Vitis)*	167	5
	*Oides Decempunctata(Vitis)*	143	6
	*Parthenolecanium Corni(Vitis)*	132	7
	*Polyphylla Laticollis Lewis(Vitis)*	120	8
	*Pseudococcus Comstocki Kuwana(Vitis)*	388	9
	*Theretra Japonica(Vitis)*	303	10
	*Vespula Flaviceps(Vitis)*	300	11
	*Xylotrechus Pyrrhoderus(Vitis)*	212	12
	*Black Rot*	1361	13
	*Esca*	1559	14
	*Grape Healthy*	541	15
	*Leaf Blight*	1220	16
Alfalfa	*Alfalfa Plant Bug(Alfalfa)*	393	1
	*Alfalfa Seed Chalcid(Alfalfa)*	273	2
	*Alfalfa Weevil(Alfalfa)*	788	3
	*Aphids(Alfalfa)*	666	4
	*Armyworm(Alfalfa)*	885	5
	*Blister Beetle(Alfalfa)*	330	6
	*Caterpillar(Alfalfa)*	595	7
	*Click Beetle(Alfalfa)*	379	8
	*Cutworm(Alfalfa)*	345	9
	*Ladybug(Alfalfa)*	616	10
	*Leaf Hopper(Alfalfa)*	544	11
	*Lygus(Alfalfa)*	647	12
	*Thrips(Alfalfa)*	612	13
	*Western Corn Rootworm(Alfalfa)*	829	14
Soybean	*Anticarsia Gemmatalis(Soybean)*	150	1
	*Aphis Glycines(Soybean)*	446	2
	*Ascotis Selenaria(Soybean)*	172	3
	*Bemisia Tabaci(Soybean)*	557	4
	*Clanis Bilineata(Soybean)*	290	5
	*Cletus Schmidti(Soybean)*	186	6
	*Etiella Zinckenella(Soybean)*	304	7
	*Helicoverpa Armigera(Soybean)*	304	8
	*Heterodera Glycines(Soybean)*	114	9
	*Leguminivora Glycinivorella(Soybean)*	317	10
	*Maruca Testulalis(Soybean)*	222	11
	*Matsumuraeses Phaseoli(Soybean)*	346	12
	*Melanagromyza Sojae(Soybean)*	121	13
	*Monolepta Hieroglyphica(Soybean)*	218	14
	*Nezara Viridula(Soybean)*	250	15
	*Odontothrips Loti(Soybean)*	268	16
	*Omiodes Indicata(Soybean)*	159	17
	*Paraluperodes Suturalis(Soybean)*	195	18
	*Piedmont Bean Bug(Soybean)*	195	19
	*Plathypena Scabra(Soybean)*	190	20
	*Riptortus Pedestris(Soybean)*	205	21
	*Spodoptera Litura(Soybean)*	246	22
	*Tetranychus Cinnabarinus(Soybean)*	268	23
	*Angular Leaf Spot*	510	24
	*Downy Mildew*	510	25
	*Soybean Healthy*	5842	26
Corn	*Agrotis Ypsilon(Corn)*	350	1
	*Anaphothrips Obscurus(Corn)*	463	2
	*Apolygus Lucorum(Corn)*	371	3
	*Chilo Suppressalis(Corn)*	315	4
	*Gryllotalpa Orientalis(Corn)*	269	5
	*Holotrichia Diomphalia(Corn)*	337	6
	*Holotrichia Oblita(Corn)*	376	7
	*Holotrichia Parallela(Corn)*	323	8
	*Laodelphax Striatellus(Corn)*	245	9
	*Mythimna Separata(Corn)*	363	10
	*Ostrinia Furnacalis(Corn)*	265	11
	*Pleonomus Canaliculatus(Corn)*	108	12
	*Porn Cricket(Corn)*	989	13
	*Peach Borer(Corn)*	414	14
	*Protaetia Brevitarsis(Corn)*	339	15
	*Puccinia Polysora*	838	16
	*Red Spider(Corn)*	317	17
	*White Margined Moth(Corn)*	88	18
	*Wireworm(Corn)*	532	19
	*Yellow Cutworm(Corn)*	287	20
Rice	*Asiatic Rice Borer(Rice)*	631	1
	*Brown Plant Hopper(Rice)*	500	2
	*Grain Spreader Thrips(Rice)*	103	3
	*Paddy Stem Maggot(Rice)*	156	4
	*Rice Bacterial Leaf Blight*	1624	5
	*Rice Blast*	2219	6
	*Rice Brown Spot*	2163	7
	*Rice Gall Midge(Rice)*	303	8
	*Rice Hispa*	565	9
	*Rice Leaf Caterpillar(Rice)*	292	10
	*Rice Leaf Roller(Rice)*	669	11
	*Rice Leaf Smut*	40	12
	*Rice Leafhopper(Rice)*	242	13
	*Rice Shell Pest(Rice)*	245	14
	*Rice Stemfly(Rice)*	221	15
	*Rice Tungro*	1308	16
	*Rice Water Weevil(Rice)*	513	17
	*Small Brown Plant Hopper(Rice)*	331	18
	*White Backed Plant Hopper(Rice)*	271	19
	*Yellow Rice Borer(Rice)*	162	20
Apple	*Adoxophyes Orana(Apple)*	285	1
	*Aphis Citricola(Apple)*	579	2
	*Carposina Sasakii(Apple)*	417	3
	*Grapholitha Molesta(Apple)*	228	4
	*Panonchus Citri(Apple)*	410	5
	*Apple Black Rot*	671	6
	*Apple Healthy*	1899	7
	*Apple Rust*	305	8
	*Apple Scab*	680	9
Wheat	*Macrosiphum Avenae(Wheat)*	544	1
	*Penthaleus Major(Wheat)*	362	2
	*Rhopalosiphum Maidis(Wheat)*	134	3
	*Rhopalosiphum Padi(Wheat)*	333	4
	*Schizaphis Graminum(Wheat)*	380	5
	*Sitobion Avenae(Wheat)*	362	6
	*Brown Rust*	1530	7
	*Wheat Healthy*	137	8
	*Yellow Rust*	1346	9
Cotton	*Adelphocoris Fasciaticollis(Cotton)*	276	1
	*Adelphocoris Lineolatus(Cotton)*	356	2
	*Adelphocoris Suturalis(Cotton)*	174	3
	*Agrotis Segetum(Cotton)*	197	4
	*Aphis Gossypii Glover(Cotton)*	306	5
	*Creontiades Dilutus(Cotton)*	163	6
	*Earias Cupreoviridis(Cotton)*	136	7
	*Helicoverpa Armigera(Cotton)*	327	8
	*Lygus Lucorum(Cotton)*	361	9
	*Lygus Pratensis(Cotton)*	192	10
	*Pectinophora Gossypiella(Cotton)*	195	11
	*Phenacoccus Solenopsis(Cotton)*	164	12
	*Spodoptera Exigua(Cotton)*	345	13
	*Spodoptera Litura(Cotton)*	182	14
	*Tetranychus Cinnabarinus(Cotton)*	294	15
	*Tetranychus Truncatus(Cotton)*	259	16
	*Thrips Tabaci(Cotton)*	217	17
Tea	*Aapiletucara Cristata(Tea)*	191	1
	*Acapimya Theae(Tea)*	155	2
	*Aleurocanthus Spiniferus(Tea)*	133	3
	*Andraca Bipunctata(Tea)*	136	4
	*Ectropis Obliqua(Tea)*	187	5
	*Empoasca Onukii(Tea)*	142	6
	*Euproctis Pseudoconspersa(Tea)*	174	7
	*Hasora Anura(Tea)*	149	8
	*Homona Coffearia(Tea)*	121	9
	*Lymantria Dispar(Tea)*	172	10
	*Parasa Lepida(Tea)*	137	11
	*Scirtothrips Dorsalis(Tea)*	218	12
	*Teinopalpus Aureus(Tea)*	176	13
	*Toxoptera Aurantii(Tea)*	192	14
	*Xyleborus Fornicatus(Tea)*	174	15
Peach	*Grapholitha Molesta(Peach)*	115	1
	*Myzus Persicae(Peach)*	236	2
	*Bacterial Spot*	2522	3
	*Peach Healthy*	405	4
Tomato	*Bacterial Spot*	2349	1
	*Early Blight*	1100	2
	*Late Blight*	2076	3
	*Leaf Mold*	1052	4
	*Mosaic Virus*	418	5
	*Septoria Leaf Spot*	1940	6
	*Spider Mites Two-Spotted Spider Mite*	1839	7
	*Target Spot*	1555	8
	*Tomato Healthy*	1761	9
	*Tomato Yellow Leaf Curl Virus*	5775	10
Potato	*Early Blight*	1100	1
	*Late Blight*	1100	2
	*Potato Healthy*	167	3
Pepper	*Bacterial Spot*	1097	1
	*Pepper Healthy*	1625	2
Strawberry	*Leaf Scorch*	1232	1
	*Strawberry Healthy*	500	2
Cherry	*Cherry Healthy*	948	1
	*Powdery Mildew*	1169	2
Raspberry	*Raspberry Healthy*	405	1
Blueberry	*Blueberry Healthy*	1657	1

*Note*: Text in italics indicates scientific names.
